# Nicotine dependence and smoking habits in patients with head and neck cancer*

**DOI:** 10.1590/S1806-37132014000300012

**Published:** 2014

**Authors:** Adriana Ávila de Almeida, Celso Muller Bandeira, Antonio José Gonçalves, Alberto José Araújo

**Affiliations:** Santa Casa de Misericórdia de São Paulo School of Medical Sciences, São Paulo, Brazil; Santa Casa de Misericórdia de São Paulo School of Medical Sciences, São Paulo, Brazil; Department of Surgery, Santa Casa de Misericórdia de São Paulo School of Medical Sciences, São Paulo, Brazil; Center for the Study and Treatment of Smoking, Thoracic Diseases Institute, Clementino Fraga Filho University Hospital, Federal University of Rio de Janeiro, Rio de Janeiro, Brazil

**Keywords:** Head and neck neoplasms, Tobacco use disorder, Smoking cessation

## Abstract

**Objective:**

To assess smoking habits and nicotine dependence (ND) in patients with head and neck cancer

**Methods:**

This study involved 71 smokers or former smokers with squamous cell carcinoma in the oral cavity, pharynx, or larynx who were treated at a university hospital in the city of São Paulo between January and May of 2010. We used the Fagerström Test for Nicotine Dependence to evaluate smoking habits and ND in the sample. Data regarding cancer treatment were collected from medical records. Depending on the variables studied, we used the chi-square test, Fisher's exact test, Student's t-test, or Spearman's correlation test.

**Results:**

Of the 71 patients, 47 (66.2%) presented with high or very high ND, 40 (56.3%) smoked more than 20 cigarettes/day, and 32 (45.1%) smoked their first cigarette within 5 min of awakening. Advanced disease stage correlated significantly with the number of cigarettes smoked per day (p = 0.011) and with smoking history (p = 0.047). We found that ND did not correlate significantly with gender, disease stage, smoking cessation, or number of smoking cessation attempts, nor did the number of cigarettes smoked per day correlate with smoking cessation or gender. Treatment for smoking cessation was not routinely offered.

**Conclusions:**

In most of the patients studied, the level of ND was high or very high. The prevalence of heavy smoking for long periods was high in our sample. A diagnosis of cancer is a motivating factor for smoking cessation. However, intensive smoking cessation treatment is not routinely offered to smoking patients diagnosed with cancer.

## Introduction

The World Health Organization considers smoking to be the leading preventable cause of death worldwide and a chronic recurrent disease caused by nicotine dependence (ND).^(^ ^1^ ^) ^Most smokers are unaware of the damage caused by chronic tobacco use, and nearly half will die of a tobacco-related disease.^(^ ^1^ ^)^ Tobacco is the most important risk factor for cancer and more than 50 diseases. More than 5,300 components, of which at least 70 are carcinogens, have been identified in tobacco smoke.^(^ ^2^ ^)^

Squamous cell carcinoma of the oral cavity, pharynx, and larynx are among the 10 most common types of cancer in men.^(^ ^3^ ^,^ ^4^ ^)^ Cancer of the oral cavity is considered a public health problem worldwide. In Brazil, it ranks fifth among men, and it is estimated that, in 2014, there will be 11,280 new cases among men and 4,010 new cases among women. Worldwide, laryngeal cancer ranks second among respiratory tract tumors, with 160,000 new cases per year. In Brazil, it ranks seventh among men, and its incidence is higher from the fourth decade of life onward. According to data from the Brazilian National Cancer Institute, in 2014, it is estimated that there will be 6,870 new cases of laryngeal cancer among men and 770 new cases of laryngeal cancer among women.^(^ ^5^ ^)^

Chief among the major risk factors are smoking and alcoholism. Studies have shown that the risk of developing cancer of the oral cavity and larynx is much higher in smokers and drinkers, depending on the duration of smoking, the number of cigarettes smoked per day, and the frequency of alcohol intake.^(^ ^3^ ^-^ ^5^ ^)^

Continuing to smoke after a diagnosis of cancer contributes to a higher risk of complications during treatment and to decreased responses to radiotherapy and chemotherapy, as well as leading to worsening of other tobacco-related diseases. Maintaining tobacco use increases the risk of recurrence and of developing a second primary tumor, with decreased quality of life and overall survival.^(^ ^6^ ^-^ ^9^ ^)^

A complex behavior, ND is influenced by genetic, social, and environmental factors, and is therefore considered a chronic disease that requires repeated interventions.^(^ ^10^ ^-^ ^12^ ^)^ Some studies have shown that, in cancer patients, ND is higher.^(^ ^13^ ^-^ ^16^ ^)^

The Fagerström Test for Nicotine Dependence (FTND) has emerged as a quick, easily administered instrument to assess ND, is widely used worldwide, and has been validated in several languages and populations.^(^ ^13^ ^,^ ^17^ ^,^ ^18^ ^)^

The assessment of ND in patients with tobacco-related cancer makes it possible to identify which patients may have difficulty in quitting smoking.^(^ ^8^ ^,^ ^18^ ^,^ ^19^ ^)^

Considering that the vast majority of patients with head and neck cancer are heavy smokers and that quitting smoking has tangible benefits in cancer treatment, we decided to conduct a study on smoking habits and ND in this population in order to provide data for interventions and approaches that are more effective for smoking cessation in cancer patients.

## Methods

This was an observational study involving patients with squamous cell carcinoma of the oral cavity, pharynx, or larynx who were treated at the Head and Neck Surgery Outpatient Clinic of the *Santa Casa de São Paulo* School of Medical Sciences, in the city of São Paulo, Brazil. The study design was submitted to and approved by the local ethics committee.

We consecutively recruited 71 subjects who met the eligibility criteria and were treated between January and May of 2010. All participants were interviewed by the same investigator and were informed of the objectives of the study. After reading the written informed consent form, they agreed to complete the questionnaire regarding ND and smoking habits.

Data regarding cancer staging and treatment were collected from medical records. Laryngectomized patients who were unable to speak completed the questionnaire by reading the questions and indicating or pointing to responses. Patients were operated on the *Santa Casa de São Paulo* over a time period ranging from one month to 15 years. We excluded patients with tumors of the nasopharynx or lip because these tumors do not have smoking as their major risk factor.

Active smokers were defined as subjects who had smoked at least 100 cigarettes in their lifetime and who, at the time of the interview, continued to smoke daily or occasionally. Smoking history was quantified by pack-years of cigarettes smoked.

For the purposes of statistical analysis, patients were separated into two groups according to their FTND scores: 0 to 5 points (very low to moderate dependence); and 6 to 10 points (high or very high dependence).

The analysis of smoking habits included questions regarding current cigarette use, age at smoking initiation, type of tobacco used, amount of tobacco used, and duration of use. Patients were asked questions regarding the number of smoking cessation attempts and the use of pharmacological treatment for smoking cessation. We assessed emotional factors (anxiety, sadness, and happiness) associated with the act of smoking, as well as behavioral factors (coffee consumption, alcohol consumption, having meals, talking on the phone, and the work environment). In addition, we obtained data regarding withdrawal symptoms after smoking cessation, contact with smokers, and number of relapses.

The presence of withdrawal symptoms was defined as the presence of one or more of the symptoms included in the definitions of tobacco withdrawal syndrome established in the Diagnostic and Statistical Manual of Mental Disorders, Fourth Edition (irritability, depression, restlessness, insomnia, anxiety, hunger, and lack of concentration).^(^ ^20^ ^)^

Patients were asked questions regarding regular alcohol use, and the Cut down, Annoyed, Guilty, and Eye-opener (CAGE) questionnaire was used to assess risky alcohol use.^(^ ^21^ ^)^ Alcohol abuse and dependence was defined as responding affirmatively to two or more questions.

For statistical purposes, patients were classified into two groups: early stages (stages I and II); and advanced stages (stages III and IV). For the same reason, patients were gathered in two groups according to the number of smoking cessation attempts: those who had not made any attempts (no attempts); and those who had made at least one attempt (one or more attempts). The responses to the first question on the FTND, i.e., the one that assesses the time to smoke the first cigarette of the day, were divided into "within 30 min of awakening" and "after 30 min of awakening".

All data were analyzed by the Statistical Package for the Social Sciences, version 13 (SPSS Inc., Chicago, IL, USA). The results were assessed by the chi-square test, Fisher's exact test, Student's t-test, or Spearman's correlation test, as appropriate. For all tests, the level of significance was set at 5%.

## Results

The present study included 71 patients with squamous cell carcinoma of the oral cavity, pharynx, or larynx. Table 1 shows the general characteristics of the patients studied. In our sample, 59 patients (83.1%) were male and 52 (73.2%) were White. The mean age of the sample was 61.4 ± 8.1 years. The mean age at smoking initiation was 15.6 ± 3.7 years (range, 6-30). The mean duration of tobacco use was 40.4 ± 10.7 years. Of the 71 patients, 50 (70.4%) had advanced stages of disease. Commercial cigarettes were the most commonly used type of tobacco (in (98.6%), 70 patients (98.6%) reported contact with smokers at home or at work, and 31 (43.7%) had never tried to quit smoking.

**Table 1:** - General characteristics of the 71 study patients.a

Characteristic	Result
Age, years^b^	61.4 ± 8.1
Gender	
Male	59 (83.1)
Female	12 (16.9)
Race	
White	52 (73.2)
Non-White	19 (26.8)
Disease site	
Larynx	38 (53.5)
Pharynx	22 (31.0)
Mouth	11 (15.5)
Disease stage	
I	15 (21.1)
II	6 (8.5)
III	17 (23.9)
IV	33 (46.5)
Cancer treatment	
Surgery + radiotherapy	10 (14.0)
Surgery + radiotherapy + chemotherapy	18 (25.4)
Surgery	27 (38.0)
Chemotherapy	1 (1.4)
Radiotherapy + chemotherapy	9 (12.7)
Radiotherapy	6 (8.5)
Age at smoking initiation, years^b^	
Overall	15.6 ± 3.7
Males	15.5 ± 3.6
Females	16.1 ± 4.5
Duration of smoking, years^b^	40.4 ± 10.7
FTND, score	5.8 ± 2.3
Low to moderate ND (0-5)	24 (33.8)
High or very high ND (6-10)	47 (66.2)
Pharmacological treatment	
Nicotine patch	2 (2.8)
Bupropion	1 (1.4)
None	68 (95.8)
Number of cigarettes smoked	
Up to 20 cigarettes/day	31 (43.7)
More than 20 cigarettes/day	40 (56.3)
Factors associated with tobacco use	
Work	66 (93.0)
Coffee	60 (84.5)
Meals	64 (90.1)
Talking on the phone	19 (26.8)
Anxiety	65 (91.5)
Happiness	58 (81.7)
Sadness	54 (76.1)
Alcohol	61 (85.9)
Number of smoking cessation attempts	
None	31 (43.7)
One	25 (35.3)
Two	5 (7.0)
Three or more	10 (14.0)
Type of cigarette smoked	
Commercial	70 (98.6)
Hand-rolled	1 (1.4)
Contact with smokers	70 (98.6)
Smoking history, pack-years^b^	60.5 ± 29.8
Daily alcohol intake	66 (93.0)

FTND : Fagerström Test for Nicotine Dependence

ND : nicotine dependence

a Values expressed as n (%), except where otherwise indicated.

b Values expressed as mean ± SD.

The mean FTND score was 5.8 ± 2.3 points for the sample as a whole. Of the respondents, 47 (66.2%) had a high or very high FTND score (Tables 1 and 2). We found that ND did not correlate significantly with gender (p = 0.970), disease stage (p = 0.620), smoking cessation (p = 0.251), or number of smoking cessation attempts (p = 0.792; Table 3).

**Table 2:** - Distribution of the 71 patients with head and neck cancer, by responses given to the Fagerström Test for Nicotine Dependence.(17)

Question	Score (points)	Response^a^
1. How soon after you wake up do you smoke your first cigarette?		
Within 5 minutes	3	32 (45.1)
6 to 30 minutes	2	23 (32.4)
31 to 60 minutes	1	5 (7.0)
After 60 minutes	0	11 (15.5)
2. Do you find it difficult to refrain from smoking in places where it is forbidden (i.e., in church, at the library, etc.)?		
Yes	1	33 (46.5)
No	0	38 (53.5)
3. Which cigarette would you hate most to give up?		
The first one in the morning	1	36 (50.7)
Any other	0	35 (49.3)
4. How many cigarettes per day do you smoke?		
31 or more	3	32 (45.1)
21 to 30	2	8 (11.2)
11 to 20	1	22 (31.0)
10 or less	0	9 (12.7)
5. Do you smoke more frequently during the first hours after waking?		
Yes	1	13 (18.3)
No	0	58 (81.7)
6. Do you smoke if you are so ill that you are in bed most of the day?		
Yes	1	42 (59.2)
No	0	29 (40.8)

a Values expressed as n (%).

**Table 3:** - Distribution of the patients with head and neck cancer by nicotine dependence and by the variables gender, disease stage, smoking cessation, and number of smoking cessation attempts.

Variable	Category	Nicotine dependence		Total	p
		Very low/low	High/very high		
Gender	Female	4 (33.3)	8 (66.7)	12 (100.0)	0.970*
	Male	20 (33.9)	39 (66.1)	59 (100.0)	
Disease stage	Early	8 (38.1)	13 (61.9)	21 (100.0)	0.620**
	Advanced	16 (32.0)	34 (68.0)	50 (100.0)	
Smoking cessation	Yes	23 (36.5)	40 (63.5)	63 (100.0)	0.251*
	No	1 (12.5)	7 (87.5)	8 (100.0)	
Number of attempts	None	11 (35.5)	20 (64.5)	31 (100.0)	0.792**
	≥ 1	13 (32.5)	27(67.5)	40 (100.0)	

* Fisher's exact test

** Chi-square test

Of the 63 patients (88.7%) who ceased smoking, 43 (60.5%) did so after diagnosis or during treatment. Of those who ceased smoking, 40 (63.5%) had a high or very high FTND score.

We found that smoking cessation did not show statistically significant correlations with level of ND (p = 0.251), with number of cigarettes smoked per day (p = 0.507), or with time to smoke the first cigarette of the day (p = 0.673; Tables 3 and 4).

**Table 4:** - Distribution of the patients with head and neck cancer by number of cigarettes smoked per day and by the variables gender, disease stage, and smoking cessation.

Variable	Category	Number of cigarettes/day		Total	p
		Up to 20 cigarettes	≥ 20 cigarettes		
Gender	Female	7 (58.3)	5 (41.7)	12 (100.0)	0.261**
	Male	24 (40.7)	35 (59.3)	59 (100.0)	
Disease stage	Early	14 (66.3)	7 (33.3)	21 (100.0)	0.011**
	Advanced	17 (34.0)	33 (66.0)	50 (100.0)	
Smoking cessation	Yes	28 (44.5)	35 (55.5)	63 (100.0)	0.507*
	No	3 (37.5)	5 (62.5)	8 (100.0)	

* Fisher's exact test

** Chi-square test

In our sample, 40 patients (56.3%) smoked more than 20 cigarettes/day, and the mean smoking history was 60.5 ± 29.8 pack-years. Although the number of cigarettes smoked per day did not correlate with the success of cessation attempts (p = 0.507) or with gender (p = 0.261), it correlated significantly with disease stage (p = 0.011; Table 4).

We found that smoking history correlated significantly with disease stage (p = 0.047) but not with time to smoke the first cigarette of the day (p = 0.270) or with smoking cessation (p = 0.960; Table 5). When we examined the association between smoking history and disease stage by using Spearman's correlation test, we found no significant correlation (r = 0.184; p = 0.125).

**Table 5:** - Distribution of the patients with head and neck cancer by smoking history and by the variables time to smoke the first cigarette of the day, disease stage, and smoking cessation.

Variable	Category	Smoking history, pack-years		Total	p
		≤ 40	> 40		
Time to smoke the first cigarette of the day	Within 30 min of awakening	13 (23.6%)	42 (76.4%)	55 (100%)	0.270**
	After 30 min of awakening	6 (37.5%)	10 (62.5%)	16 (100%)	
Disease stage	Early	9 (42.9%)	12 (57.1%)	21 (100%)	0.047**
	Advanced	10 (20.0%)	40 (80.0%)	50 (100%)	
Smoking cessation	Yes	17 (27.0%)	46 (73.0%)	63 (100%)	0.960*
	No	2 (25.0%)	6 (75.0%)	8 (100%)	

* Fisher's exact test

Of the 71 study participants, only 3 used smoking cessation medication. Two were successfully treated with nicotine replacement therapy, and one used bupropion but relapsed. Of the 61 subjects who did not use medication, 43 (70.5%) reported withdrawal symptoms during smoking cessation, and, of those, 29 (46%) presented with high or very high dependence. Anxiety was the most commonly reported withdrawal symptom among those who ceased smoking.

Regarding alcohol use, 66 subjects (93%) responded affirmatively to at least two of the four questions on the CAGE questionnaire.

The workplace, meal times, and coffee or alcohol consumption were considered triggers for smoking for the vast majority of respondents, whereas anxiety was the emotional factor most commonly associated with the act of smoking (Table 1).

## Discussion

Squamous cell carcinoma of the head and neck is one of the most common types of cancer worldwide, and the prevalence of heavy smoking is high in this group.^(^ ^3^ ^,^ ^13^ ^,^ ^15^ ^)^ Considering that continuing to smoke after a diagnosis of cancer contributes to a higher risk of complications during treatment and of development of a second primary tumor,^(^ ^6^ ^-^ ^8^ ^,^ ^22^ ^)^ the present study was designed to assess ND and smoking habits in this population.

The assessment of ND in cancer patients can contribute to the development of an approach that is more effective and has longer-term effects in terms of smoking cessation.^(^ ^8^ ^,^ ^15^ ^,^ ^22^ ^,^ ^23^ ^) ^Although the FTND does not assess motivational or emotional components, it is able to predict withdrawal symptoms and guide the pharmacological treatment during smoking cessation.^(^ ^22^ ^,^ ^24^ ^)^

In our sample, we found 47 subjects (66.2%) with high or very high ND. These results confirm the findings of other studies that also reported high ND in head and neck cancer patients.^(^ ^13^ ^,^ ^19^ ^)^ We found that ND did not correlate with gender, disease stage, smoking cessation, or number of smoking cessation attempts. We also found that the number of cigarettes smoked per day did not correlate significantly with smoking cessation or gender.

Questions 1 and 4 on the FTND are the ones of greatest importance.^(^ ^17^ ^,^ ^24^ ^)^ In our sample, 55 subjects (77.5%) reported smoking their first cigarette within 30 min of awakening, and 32 (45.1%) smoked more than 30 cigarettes per day. These data are consistent with those of a study^(^ ^24^ ^)^ of 301 regular smokers, which showed that a shorter time to smoke the first cigarette of the day and a greater number of cigarettes smoked per day translated into a greater level of ND.

Many patients continue to smoke even though they are aware that smoking is a risk factor for morbidity and a factor increasing morbidity during treatment.^(^ ^8^ ^,^ ^9^ ^,^ ^13^ ^-^ ^16^ ^,^ ^22^ ^)^ However, in our sample, 63 patients (88.7%) ceased tobacco use. Of those, only 2 received pharmacological treatment (nicotine patch). This result confirms literature reports that smoking after a cancer diagnosis is yet to be routinely addressed as a disease requiring treatment.^(^ ^8^ ^,^ ^9^ ^,^ ^16^ ^,^ ^23^ ^)^

Our results for age at smoking initiation were similar to those reported in the literature-90% of smokers start smoking before 19 years of age. Earlier age at smoking initiation is known to translate into a greater chance of becoming dependent.^(^ ^25^ ^)^ In our sample, the mean age at smoking initiation was 15.6 ± 3.7 years (range, 6-30 years).

Smoking is a recurrent disease, and multiple cessation attempts are common until permanent cessation success is achieved.^(^ ^10^ ^,^ ^26^ ^)^ In our sample, 40 subjects had made at least one attempt.

The mean smoking history was 60.5 ± 29.8 pack-years. There was a significant association between smoking history and disease stage (p = 0.047; chi-square test). However, when we examined the association between smoking history and disease stage by using Spearman's correlation test, we found no significant correlation (r = 0.184; p = 0.125). This difference can be explained by the fact that Spearman's correlation test requires linear correlation of distribution, which cannot be seen when considering subgroups of early disease patients and advanced disease patients.

Analysis of smoking history revealed that the number of cigarettes smoked ever did not affect the success of cessation attempts (p = 0.960).

In our study, 66 subjects (93%) were considered alcohol dependent by the CAGE questionnaire.^(^ ^21^ ^)^ In addition, alcohol was reported as a trigger for tobacco use by 61 subjects (85.9%), which is a cause for concern, especially in cases of alcohol abuse and smoking. Because smoking and alcoholism are important risk factors, they should be treated concurrently in patients with head and neck cancer.^(^ ^3^ ^,^ ^4^ ^,^ ^7^ ^,^ ^13^ ^,^ ^22^ ^)^

The time of diagnosis and the course of treatment are a great opportunity for promoting behavioral changes, and we should encourage cessation of tobacco and alcohol use; however, this approach should be tailored to each patient. ^(^ ^13^ ^-^ ^15^ ^,^ ^22^ ^)^ The basic model of intervention should progressively increase in intensity according to the needs of the patient, also including adjuvant pharmacological treatment.^(^ ^8^ ^,^ ^12^ ^,^ ^13^ ^,^ ^19^ ^,^ ^22^ ^,^ ^23^ ^)^

Cancer treatment is frequently accompanied by the discomfort of surgery, radiotherapy, and/or chemotherapy. These factors alone could contribute to patients experiencing anxiety or mood swings, or an exacerbation of these problems, which could add to or be mistaken for the withdrawal symptoms due to concurrent cessation of tobacco and (in many cases) alcohol use.^(^ ^14^ ^)^

Smoking cessation interventions in oncology should focus not only on the risks of continued tobacco use, but also, and mainly, on supporting long-term abstinence and reducing relapse risk factors, which are very common in this population. ^(^ ^8^ ^,^ ^9^ ^,^ ^23^ ^,^ ^27^ ^)^

It is essential that oncologists understand that smoking and alcoholism are diseases that should be approached and treated properly and are components of the cancer treatment process as a whole.^(^ ^9^ ^,^ ^16^ ^,^ ^23^ ^,^ ^27^ ^)^

The present study has some limitations to be considered. First, although there are scales for assessing withdrawal symptoms, we concluded that it would be difficult to apply them because of the long period of continued abstinence of the former smokers in the present study. Second, although the literature reports similar results for the FTND in smokers and former smokers, it is possible that the long interval between smoking cessation and the administration of the questionnaire influenced patients' responses, thereby reducing the sensitivity of the test.^(^ ^28^ ^-^ ^30^ ^)^

In conclusion, the level of ND was high or very high in most patients in our study, and the prevalence of heavy smoking (more than 20 cigarettes/day) for long periods was high. Although most respondents were successful in smoking cessation, withdrawal symptoms were common and pharmacological treatment was not routinely offered. There was a significant association between smoking history and advanced disease stage. The approach to head and neck cancer patients who smoke is a window of opportunity for offering smoking cessation treatment. A diagnosis of cancer is a motivator for smoking cessation. However, intensive smoking cessation treatment is not routinely performed.
