# Supplementary material for: Nicotine dependence and smoking habits in patients with head and neck cancer
Source: J Bras Pneumol. 2014 May-Jun;40(3):286–93. doi: 10.1590/S1806-37132014000300012 (PMC4109201; doi:10.1590/S1806-37132014000300012)
Supplement: Translation

# Translation: Dependência nicotínica e perfil tabágico em pacientes com câncer de cabeça e pescoço*

**Authors:** Adriana Ávila de Almeida, Celso Muller Bandeira, Antonio José Gonçalves, Alberto José Araújo

**Affiliations:** Faculdade de Ciências Médicas, Santa Casa de Misericórdia de São Paulo, Santa Casa de Misericórdia de São Paulo, São Paulo (SP) Brasil; Faculdade de Ciências Médicas, Santa Casa de Misericórdia de São Paulo, São Paulo (SP) Brasil; Departamento de Cirurgia, Faculdade de Ciências Médicas, Santa Casa de Misericórdia de São Paulo, São Paulo (SP) Brasil; Médico Pneumologista Coordenador. Núcleo de Estudos e Tratamento do Tabagismo, Instituto de Doenças do Tórax, Hospital Universitário Clementino Fraga Filho, Universidade Federal do Rio de Janeiro, Rio de Janeiro (RJ) Brasil

**Keywords:** Neoplasias de cabeça e pescoço, Transtorno por uso de tabaco, Abandono do hábito de fumar

## Abstract

**Objetivo:**

Avaliar o perfil tabágico (PT) e a dependência nicotínica (DN) em pacientes com câncer de cabeça e pescoço.

**Métodos:**

Estudo realizado com 71 pacientes portadores de carcinoma epidermoide de cavidade oral, faringe e laringe, tabagistas ou ex-tabagistas, atendidos em um hospital universitário da cidade de São Paulo entre janeiro e maio de 2010. Utilizou-se o Teste de Fagerström para Dependência de Nicotina para avaliar PT e DN na amostra. Informações sobre o tratamento oncológico foram coletadas dos prontuários. Foram utilizados os testes do qui-quadrado, exato de Fisher ou t de Student, assim como o teste de correlação de Spearman conforme as variáveis estudadas.

**Resultados:**

Dos 71 pacientes, 47 (66,2%) apresentavam DN elevada ou muito elevada, 40 (56,3%) fumavam mais de 20 cigarros/dia, e 32 (45,1%) fumavam seu primeiro cigarro até 5 min após acordar. Houve associações significativas do estádio avançado da doença com a quantidade de cigarros fumados por dia (p = 0,011) e a carga tabágica (p = 0,047). Não houve diferenças significativas da DN em relação a sexo, estádio da doença, cessação tabágica ou tentativas anteriores de cessação, nem como do número de cigarros fumados ao dia em relação a cessação ou sexo. O tratamento do tabagismo não foi rotineiramente oferecido.

**Conclusões:**

A DN foi elevada ou muito elevada na maioria dos pacientes estudados. A prevalência de grandes fumantes por longos períodos foi alta em nossa amostra. O diagnóstico de câncer é um fator motivador para a cessação tabágica; entretanto, o tratamento intensivo do tabagismo ainda não é rotineiramente oferecido a fumantes diagnosticados com câncer.

## Introdução

O tabagismo é considerado pela Organização Mundial de Saúde a principal causa de morte evitável em todo o mundo e uma doença crônica e recorrente causada pela dependência nicotínica (DN).^(^ ^1^ ^)^ A maioria dos usuários desconhece os danos causados pelo uso crônico do tabaco, e quase a metade morrerá de alguma doença relacionada ao seu uso.^(^ ^1^ ^)^ O tabaco é o mais importante fator de risco para o câncer e para mais de 50 doenças. Na fumaça do tabaco já foram identificadas mais de 5.300 componentes, dentre eles, ao menos 70 carcinógenos.^(^ ^2^ ^)^

O carcinoma epidermoide de cavidade oral, faringe e laringe estão entre os 10 tipos de câncer mais frequentes entre os homens.^(^ ^3^ ^,^ ^4^ ^)^ O câncer de cavidade oral é considerado um problema de saúde pública em todo o mundo. No Brasil, ocupa a quinta posição entre os homens, com estimativas para 2014 de 11.280 casos novos entre os homens e de 4.010 para as mulheres. No mundo, o câncer de laringe é o segundo entre os tumores do aparelho respiratório, com 160.000 casos novos por ano. No Brasil, ocupa a sétima posição entre os homens, com incidência maior a partir da quarta década de vida. Segundo dados do Instituto Nacional do Câncer de 2014, estimam-se 6.870 casos novos de câncer de laringe entre os homens e 770 entre as mulheres.^(^ ^5^ ^)^

Entre os principais fatores de risco destacam-se o tabagismo e o etilismo. Estudos mostram um risco muito maior de se desenvolver câncer de cavidade oral e laringe em indivíduos tabagistas e etilistas, em função do tempo que se fuma, do número de cigarros fumados ao dia e da frequência de ingestão de etílicos.^(^ ^3^ ^-^ ^5^ ^)^

Continuar fumando após o diagnóstico do câncer contribui para um maior risco de complicações durante o tratamento, diminuição das respostas a radioterapia e quimioterapia, além de possibilitar o agravamento de outras doenças relacionadas ao tabaco. A manutenção do uso do tabaco aumenta o risco de recidiva e do aparecimento de um segundo tumor primário, com diminuição da qualidade de vida e sobrevida global.^(^ ^6^ ^-^ ^9^ ^)^

A DN é um comportamento complexo, influenciado por fatores genéticos, sociais e ambientais e, por isso, é considerada uma doença crônica que necessita de repetidas intervenções.^(^ ^10^ ^-^ ^12^ ^)^ Alguns estudos têm mostrado que, em pacientes com câncer, a DN é mais elevada.^(^ ^13^ ^-^ ^16^ ^)^

O Teste de Fagerström para Dependência de Nicotina (TFDN) surgiu como um instrumento rápido e de fácil aplicação para a avaliação da DN, sendo amplamente utilizado no mundo e já validado em diversos idiomas e populações.^(^ ^13^ ^,^ ^17^ ^,^ ^18^ ^)^

A avaliação da DN em pacientes com câncer atribuído ao tabaco possibilita identificar quais pacientes poderão apresentar dificuldades para deixar de fumar.^(^ ^8^ ^,^ ^18^ ^,^ ^19^ ^)^

Considerando-se que a imensa maioria dos pacientes com câncer de cabeça e pescoço são grandes fumantes e que parar de fumar traz benefícios concretos no tratamento oncológico, decidimos realizar um estudo sobre o perfil e a DN nessa população com o intuito de promover informações para intervenções e abordagens mais eficazes na cessação tabágica em pacientes oncológicos.

## Métodos

Estudo observacional envolvendo pacientes portadores de carcinoma epidermoide de cavidade oral, faringe e laringe atendidos no Ambulatório de Cirurgia de Cabeça e Pescoço da Faculdade de Ciências Médicas da Santa Casa de São Paulo, em São Paulo (SP). O projeto foi submetido e aprovado pelo comitê de ética da instituição. 

Foram recrutados consecutivamente 71 indivíduos que preenchiam os critérios de elegibilidade atendidos entre janeiro e maio de 2010. Todos os participantes foram entrevistados por um mesmo examinador e informados sobre os objetivos do estudo. Após lerem o termo de consentimento livre e esclarecido, concordaram em responder ao questionário sobre DN e perfil tabágico.

Informações sobre o estadiamento e o tratamento oncológico foram coletados dos prontuários médicos. Os pacientes laringectomizados não falantes preencheram o questionário mediante a leitura das perguntas e sinalização ou apontamento das respostas. Os pacientes foram operados na Santa Casa de São Paulo em um intervalo de tempo que variou de um mês a 15 anos. Foram excluídos pacientes portadores de tumores de rinofaringe ou de lábio por não terem o tabagismo como fator de risco principal.

Foram considerados tabagistas ou fumantes ativos os indivíduos que fumaram no mínimo 100 cigarros na vida e que, durante a entrevista, permaneciam fumando diariamente ou ocasionalmente. A quantificação da carga tabágica foi feita com o cálculo de maços-ano.

Para fins de análise estatística, os indivíduos foram separados em dois grupos de acordo com a pontuação do TFDN: de 0 a 5 pontos (dependência muito baixa a moderada) e de 6 a 10 pontos (dependência elevada ou muito elevada). 

Na analise do perfil tabágico, foram incluídas perguntas referentes ao uso atual do cigarro, idade de início do tabagismo, tipo de consumo de tabaco, sua quantidade e tempo de uso. Foram feitas perguntas relativas ao número de tentativas anteriores de cessação tabágica e auxílio medicamentoso para tanto. Foram avaliados os fatores emocionais (ansiedade, tristeza e alegria) associados ao ato de fumar, assim como fatores comportamentais (café, álcool, refeições, falar ao telefone e ambiente de trabalho). Também foram obtidas informações sobre sintomas de abstinência após a cessação, contato com fumantes e número de recaídas.

Em relação à presença de sintomas de abstinência, adotamos como critério positivo a presença de um ou mais dos sintomas incluídos nas definições de síndrome de abstinência do tabaco pelo Manual Diagnóstico e Estatístico de Transtornos Mentais, 4^a^ edição (irritabilidade, depressão, agitação, insônia, ansiedade, fome e falta de concentração).^(^ ^20^ ^)^

Foram feitas perguntas sobre o uso regular de álcool, e o instrumento utilizado para avaliar o consumo de risco pelo uso do álcool foi o questionário *Cut down, Annoyed, Guilty, and Eye-opener* (CAGE).^(^ ^21^ ^)^ Consideramos positivo para abuso e dependência do álcool quem respondesse afirmativamente a duas ou mais questões.

Para fins estatísticos, os pacientes foram classificados em dois grupos: estágios iniciais (estádios I e II) e estágios avançados (estádios III e IV). Pelo mesmo motivo, em relação às tentativas anteriores de cessação, reunimos os indivíduos em dois grupos: os que não haviam feito tentativa alguma anteriormente (nenhuma tentativa anterior) e os que haviam feito ao menos uma tentativa (uma ou mais tentativas anteriores). Em relação à primeira pergunta do TFDN, que avalia o primeiro cigarro fumado do dia, as respostas foram divididas em "até 30 min" e "após 30 min".

As informações foram analisadas pelo programa *Statistical Package for the Social Sciences*, versão 13 (SPSS Inc., Chicago, IL, EUA). Os resultados foram avaliados pelos testes do qui-quadrado, exato de Fisher e t de Student, assim como pelo teste de correlação de Spearman. Todos os testes adotaram um nível de significância de 5%.

## Resultados

Foram incluídos 71 pacientes portadores de carcinoma epidermoide de cavidade oral, faringe e laringe no presente estudo. A Tabela 1 mostra as características gerais dos pacientes estudados. Em nossa amostra, 59 pacientes (83,1%) eram do sexo masculino e 52 (73,2%) eram da raça branca. A média de idade da amostra foi de 61,4 ± 8,1 anos. A média de idade do início do uso do tabaco foi de 15,6 ± 3,7 anos (variação, 6-30). A duração média do consumo do tabaco foi de 40,4 ± 10,7 anos. Dos 71 pacientes, 50 (70,4%) estavam em estágios avançados da doença. O cigarro industrializado foi o tipo de tabaco mais consumido (98,6%), 70 pacientes (98,6%) referiram convívio com fumantes em casa ou no trabalho, e 31 (43,7%) nunca haviam tentado parar de fumar anteriormente.

**Tabela 1:** Características gerais dos 71 pacientes do estudo.a

Características	Resultados
Idade, anos^b^	61,4 ± 8,1
Sexo	
Masculino	59 (83,1)
Feminino	12 (16,9)
Raça	
Branca	52 (73,2)
Não branca	19 (26,8)
Sítio da doença	
Laringe	38 (53,5)
Faringe	22 (31,0)
Boca	11 (15,5)
Estádio da doença	
I	15 (21,1)
II	6 (8,5)
III	17 (23,9)
IV	33 (46,5)
Tratamento oncológico	
Cirurgia + radioterapia	10 (14,0)
Cirurgia + radioterapia + quimioterapia	18 (25,4)
Cirurgia	27 (38,0)
Quimioterapia	1 (1,4)
Radioterapia + quimioterapia	9 (12,7)
Radioterapia	6 (8,5)
Idade do início do uso de tabaco, anos^b^	
Geral	15,6 ± 3,7
Homens	15,5 ± 3,6
Mulheres	16,1 ± 4,5
Duração do tabagismo, anosb	40,4 ± 10,7
TFDN, pontos	5,8 ± 2,3
DN baixa a moderada (0-5)	24 (33,8)
DN elevada ou muito elevada (6-10)	47 (66,2)
Tratamento medicamentoso	
Adesivo de nicotina	2 (2,8)
Bupropiona	1 (1,4)
Nenhum	68 (95,8)
Quantidade de cigarros fumados	
Até 20 cigarros/dia	31 (43,7)
Acima de 20 cigarros/dia	40 (56,3)
Fatores associados ao uso do tabaco	
Trabalho	66 (93,0)
Café	60 (84,5)
Refeições	64 (90,1)
Falar ao telefone	19 (26,8)
Ansiedade	65 (91,5)
Alegria	58 (81,7)
Tristeza	54 (76,1)
Álcool	61 (85,9)
Tentativas anteriores de cessação	
Nenhuma	31 (43,7)
Uma	25 (35,3)
Duas	5 (7,0)
Três ou mais	10 (14,0)
Tipo de cigarro consumido	
Industrializado	70 (98,6)
Artesanal	1 (1,4)
Convívio com fumantes	70 (98,6)
Carga tabágica, maços-anob	60,5 ± 29,8
Ingestão diária de álcool	66 (93,0)

A média do TFDN foi de 5,8 ± 2,3 pontos para toda a amostra. Dentre os participantes, 47 (66,2%) apresentaram TFDN elevado ou muito elevado (Tabelas 1 e 2). Em relação à DN, não foram observadas diferenças significativas em comparação com as seguintes variáveis: sexo (p = 0,970), estágio da doença (p = 0,620), cessação (p = 0,251) ou tentativas anteriores de cessação (p = 0,792; Tabela 3).

**Tabela 2:** Distribuição dos 71 pacientes com câncer de cabeça e pescoço segundo o Teste de Fagerström para Dependência de Nicotina(17) segundo as respostas fornecidas.

Perguntas	Pontos	Respostas^a^
1. Quanto tempo depois de acordar você fuma seu primeiro cigarro?		
Dentro de 5 minutos	3	32 (45,1)
Entre 6 e 30 minutos	2	23 (32,4)
Entre 31 e 60 minutos	1	5 (7,0)
Após 60 minutos	0	11 (15,5)
2. Você acha difícil não fumar em lugares proibidos como igrejas, bibliotecas, etc.?		
Sim	1	33 (46,5)
Não	0	38 (53,5)
3. Qual o cigarro do dia lhe traz mais satisfação?		
O primeiro da manhã	1	36 (50,7)
Outros	0	35 (49,3)
4. Quantos cigarros você fuma por dia?		
31 ou mais	3	32 (45,1)
21 a 30	2	8 (11,2)
11 a 20	1	22 (31,0)
10 ou menos	0	9 (12,7)
5. Você fuma mais frequentemente pela manhã?		
Sim	1	13 (18,3)
Não	0	58 (81,7)
6. Você fuma mesmo doente, quando precisa ficar na cama a maior parte do dia?		
Sim	1	42 (59,2)
Não	0	29 (40,8)

a Valores expressos em n (%).

**Tabela 3:** Distribuição dos pacientes com câncer de cabeça e pescoço segundo a dependência nicotínica e as variáveis sexo, estágio da doença, cessação tabágica e tentativas anteriores de cessação.

Variáveis	Categorias	Dependência Nicotínica		Total	p
		Muito baixa/baixa	Elevada/muito elevada		
Sexo	Feminino	4 (33,3)	8 (66,7)	12 (100,0)	0,970*
	Masculino	20 (33,9)	39 (66,1)	59 (100,0)	
Estágio da doença	Inicial	8 (38,1)	13 (61,9)	21 (100,0)	0,620**
	Avançado	16 (32,0)	34 (68,0)	50 (100,0)	
Cessação	Sim	23 (36,5)	40 (63,5)	63 (100,0)	0,251*
	Não	1 (12,5)	7 (87,5)	8 (100,0)	
Tentativas anteriores	Nenhuma	11 (35,5)	20 (64,5)	31 (100,0)	0,792**
	≥ 1	13 (32,5)	27(67,5)	40 (100,0)	

* Teste exato de Fisher

** Teste do qui-quadrado.

Dentre os 63 pacientes (88,7%) que cessaram o tabagismo, 43 (60,5%) o fizeram após o diagnóstico ou durante o tratamento. Entre os que cessaram, 40 (63,5%) apresentavam TFDN elevado ou muito elevado. 

Não foram observadas diferenças estatisticamente significativas quando comparamos a cessação tabágica com o grau de DN (p = 0,251), com a quantidade de cigarros fumados ao dia (p = 0,507) ou com o tempo para fumar o primeiro cigarro (p = 0,673; Tabelas 3 e 4).

**Tabela 4:** Distribuição dos pacientes com câncer de cabeça e pescoço segundo a quantidade de cigarros fumados ao dia e as variáveis sexo, estágio da doença e cessação tabágica.

Variáveis	Categorias	Quantidade de cigarros/dia		Total	p
		Até 20 cigarros	≥ 20 cigarros		
Sexo	Feminino	7 (58,3)	5 (41,7)	12 (100,0)	0,261**
	Masculino	24 (40,7)	35 (59,3)	59 (100,0)	
Estágio	Inicial	14 (66,3)	7 (33,3)	21 (100,0)	0,011**
	Avançado	17 (34,0)	33 (66,0)	50 (100,0)	
Cessação	Sim	28 (44,5)	35 (55,5)	63 (100,0)	0,507*
	Não	3 (37,5)	5 (62,5)	8 (100,0)	

* Teste exato de Fisher

** Teste do qui-quadrado.

Em nossa amostra, 40 pacientes (56,3%) fumavam acima de 20 cigarros/dia, e a média da carga tabágica foi de 60,5 ± 29,8 maços-ano. Não houve associação entre a quantidade de cigarros fumados com o sucesso da tentativa de cessação (p = 0,507) ou com o sexo (p = 0,261); contudo, essa associação foi significante quando comparada com os estágios da doença (p = 0,011; Tabela 4).

Encontramos diferença significativa quando comparamos a carga tabágica com os estágios da doença (p = 0,047); porém, não houve significância em relação ao primeiro cigarro fumado no dia (p = 0,270) e à cessação (p = 0,960; Tabela 5). Quando utilizamos o teste de correlação de Spearman entre a carga tabágica e o estadiamento da doença, não encontramos uma correlação significativa (r = 0,184; p = 0,125).

**Tabela 5:** Distribuição dos pacientes com câncer de cabeça e pescoço segundo a carga tabágica e as variáveis primeiro cigarro fumado no dia, estágio da doença e cessação tabágica.

Variáveis	Categorias	Carga tabágica, anos/maço		Total	p
		≤ 40	> 40		
Primeiro cigarro	Até 30 min	13 (23,6%)	42 (76,4%)	55 (100%)	0,270**
	Após 30 min	6 (37,5%)	10 (62,5%)	16 (100%)	
Estágio	Inicial	9 (42,9%)	12 (57,1%)	21 (100%)	0,047**
	Avançado	10 (20,0%)	40 (80,0%)	50 (100%)	
Cessação	Sim	17 (27,0%)	46 (73,0%)	63 (100%)	0,960*
	Não	2 (25,0%)	6 (75,0%)	8 (100%)	

* Teste exato de Fisher

** Teste do qui-quadrado.

Entre os 71 participantes do estudo, apenas 3 usaram medicação para tratar o tabagismo. Dois foram tratados com terapia de reposição de nicotina com sucesso e um utilizou bupropiona mas teve recaída. Dentre os 61 indivíduos que não utilizaram medicação, 43 (70,5%) relataram algum sintoma de abstinência durante a cessação e, dentre esses, 29 (46%) apresentavam dependência elevada ou muito elevada. A ansiedade foi o sintoma de abstinência mais frequente entre os que cessaram.

Em relação ao consumo de álcool, 66 indivíduos (93%) responderam afirmativamente ao menos duas das quatro questões do questionário CAGE.

Foram considerados gatilhos associados ao hábito de fumar o local de trabalho, o período das refeições e o consumo café ou álcool para a grande maioria dos entrevistados, enquanto a ansiedade foi o fator emocional mais associado ao ato de fumar (Tabela 1).

## Discussão

O carcinoma epidermoide de cabeça e pescoço é um dos tipos mais frequentes de câncer no mundo, e a prevalência de grandes fumantes nesse grupo é alta.^(^ ^3^ ^,^ ^13^ ^,^ ^15^ ^)^ Considerando-se que continuar fumando após o diagnóstico de câncer contribui para um maior risco de complicações no tratamento e de aparecimento de um segundo tumor primário,^(^ ^6^ ^-^ ^8^ ^,^ ^22^ ^)^ o presente estudo foi idealizado para a avaliação da DN e do perfil tabágico nessa população.

A avaliação da DN em pacientes oncológicos pode contribuir para uma abordagem mais efetiva e com resultados mais prolongados em relação à cessação tabágica.^(^ ^8^ ^,^ ^15^ ^,^ ^22^ ^,^ ^23^ ^)^ Apesar de não avaliar componentes motivacionais ou emocionais, o TFDN é capaz de prever os sintomas de abstinência e direcionar o tratamento medicamentoso durante a cessação.^(^ ^22^ ^,^ ^24^ ^)^

Em nossa casuística, observamos 47 indivíduos (66,2%) com DN elevada ou muito elevada. Esses resultados confirmam os achados de outros estudos que também encontraram elevada DN nessa mesma população.^(^ ^13^ ^,^ ^19^ ^)^ Em relação à DN, não houve diferenças quanto ao sexo, estádio da doença, cessação ou tentativas anteriores de cessação. Não foram encontradas associações significativas da quantidade de cigarros fumados ao dia com cessação ou sexo.

As perguntas de número 1 e 4 do TFDN são as de maior peso.^(^ ^17^ ^,^ ^24^ ^)^ Na nossa casuística, 55 indivíduos (77,5%) relataram fumar o primeiro cigarro em até 30 min após acordar, e 32 (45,1%) fumavam mais de 30 cigarros ao dia. Esses dados estão de acordo com um estudo^(^ ^24^ ^)^ com 301 fumantes regulares, que mostrou que quanto menor for o tempo para fumar o primeiro cigarro e quanto maior o número de cigarros fumados ao dia, maior é o grau de DN. 

Muitos pacientes permanecem fumando embora sejam informados sobre o tabagismo como um fator de risco e de aumento da morbidade durante o tratamento.^(^ ^8^ ^,^ ^9^ ^,^ ^13^ ^-^ ^16^ ^,^ ^22^ ^)^ Contudo, em nossa amostra, 63 pacientes (88,7%) cessaram o uso do tabaco. Dentre esses, apenas 2 utilizaram tratamento medicamentoso (adesivo de nicotina). Esse resultado comprova os dados de literatura que demonstram que o tabagismo após o diagnóstico de câncer ainda não é rotineiramente abordado como uma doença que necessita de tratamento.^(^ ^8^ ^,^ ^9^ ^,^ ^16^ ^,^ ^23^ ^)^

Em relação à idade de início do tabagismo, encontramos números semelhantes aos da literatura mundial - 90% de fumantes iniciando o tabagismo antes dos 19 anos de idade. Sabe-se que quanto mais precoce for o início do uso do tabaco, maiores serão as chances de o fumante tornar-se dependente.^(^ ^25^ ^)^ Em nossa casuística, a média de idade de início do uso do tabaco foi de 15,6 ± 3,7 anos, variando de 6 a 30 anos. 

O tabagismo é uma doença recorrente e são comuns as múltiplas tentativas de cessação até a parada definitiva.^(^ ^10^ ^,^ ^26^ ^)^ Em nossa amostra, 40 indivíduos já haviam feito ao menos uma tentativa.

A média da carga tabágica foi de de 60,5 ± 29,8 maços-ano. Houve uma associação significativa entre a carga tabágica e o estágio da doença (p = 0,047; teste do qui-quadrado). No entanto, quando analisamos a correlação entre a carga tabágica e o estadiamento, não encontramos uma correlação significativa pelo teste de correlação de Spearman (r = 0,184; p = 0,125). Essa diferença pode ser justificada pelo fato de que esse teste necessita de uma correlação linear de distribuição, a qual não pode ser observada quando consideramos os subgrupos de doença em estágio inicial e em estágio avançado.

Ao analisar a carga tabágica, verificou-se que a quantidade de cigarros fumados durante toda a vida não interferiu no sucesso da tentativa de cessação (p = 0,960).

Em nosso estudo, 66 indivíduos (93%) foram considerados dependentes de álcool pelo questionário CAGE.^(^ ^21^ ^)^ O álcool também foi apontado como um gatilho para o uso do tabaco por 61 indivíduos (85,9%), o que é um fator preocupante especialmente quando há abuso de álcool concomitante ao tabagismo. Por representarem fatores de risco importantes, o tratamento do tabagismo deve ser associado ao do etilismo em pacientes com câncer de cabeça e pescoço.^(^ ^3^ ^,^ ^4^ ^,^ ^7^ ^,^ ^13^ ^,^ ^22^ ^)^

O momento do diagnóstico ou o decorrer do tratamento são uma grande oportunidade para promover mudanças de comportamento e devemos incentivar a cessação tanto do tabaco quanto do álcool; entretanto, essa abordagem deve ser adaptada para cada paciente.^(^ ^13^ ^-^ ^15^ ^,^ ^22^ ^)^ O modelo básico de intervenção deve aumentar progressivamente de intensidade de acordo com as necessidades do paciente, incluindo-se, também, o tratamento medicamentoso adjuvante.^(^ ^8^ ^,^ ^12^ ^,^ ^13^ ^,^ ^19^ ^,^ ^22^ ^,^ ^23^ ^)^

O tratamento oncológico não raramente está acompanhado do desconforto do procedimento cirúrgico, da radioterapia e/ou da quimioterapia. Esses fatores por si só já poderiam contribuir para o surgimento ou exacerbação de quadros de ansiedade ou de alteração do humor, que poderiam ser adicionados ou confundidos com os sintomas de abstinência decorrentes da cessação simultânea do uso do tabaco e, muitas vezes, do álcool.^(^ ^14^ ^)^

As intervenções para a cessação do tabagismo em oncologia devem estar voltadas não somente para os riscos que advêm da permanência do consumo do tabaco, mas principalmente para o apoio à abstinência prolongada e para a diminuição dos riscos de recaída, os quais são muito comuns nessa população.^(^ ^8^ ^,^ ^9^ ^,^ ^23^ ^,^ ^27^ ^)^

É essencial que o oncologista compreenda que o tabagismo, assim como o etilismo, são doenças que devem ser abordadas e tratadas de maneira adequada e que fazem parte do tratamento oncológico como um todo.^(^ ^9^ ^,^ ^16^ ^,^ ^23^ ^,^ ^27^ ^)^

O presente estudo tem algumas limitações a serem consideradas. Em primeiro lugar, apesar da existência de escalas para a avaliação de sintomas de abstinência, concluímos que seria difícil aplicá-las devido ao longo intervalo de tempo de abstinência continuada nos ex-fumantes do presente estudo. Em segundo lugar, apesar de a literatura relatar resultados semelhantes sobre a aplicação do TFDN em fumantes e ex-fumantes, é possível que o longo intervalo de tempo entre a cessação e a aplicação do questionário possa ter influenciado as respostas dos pacientes, diminuindo assim a sensibilidade do teste.^(^ ^28^ ^-^ ^30^ ^)^

Concluímos que a DN foi elevada ou muito elevada na maioria dos pacientes no presente estudo, com uma alta prevalência de pacientes que fumaram mais de 20 cigarros/dia e por um longo período. Apesar de a maioria dos entrevistados terem conseguido a cessação, os sintomas de abstinência foram frequentes e o tratamento medicamentoso não foi rotineiramente oferecido. Houve uma associação significativa entre a carga tabágica e o estágio avançado da doença. A abordagem do paciente com câncer de cabeça pescoço que fuma é uma janela de oportunidades para oferecer o tratamento do tabagismo. O diagnóstico de câncer é um fator motivador para a cessação tabágica; entretanto, o tratamento intensivo do tabagismo ainda não é rotineiramente realizado.
